# Post-COVID Pain Is Not Associated with Inflammatory Polymorphisms in People Who Had Been Hospitalized by COVID-19

**DOI:** 10.3390/jcm11195645

**Published:** 2022-09-25

**Authors:** César Fernández-de-las-Peñas, Rocco Giordano, Gema Díaz-Gil, Francisco Gómez-Esquer, Silvia Ambite-Quesada, Maria A. Palomar-Gallego, Lars Arendt-Nielsen

**Affiliations:** 1 Department of Physical Therapy, Occupational Therapy, Rehabilitation and Physical Medicine, Universidad Rey Juan Carlos, 28922 Alcorcón, Spain; 2 Center for Neuroplasticity and Pain (CNAP), SMI, Department of Health Science and Technology, School of Medicine, Aalborg University, 9220 Aalborg, Denmark; 3 Research group GAMDES, Department of Basic Health Sciences, Universidad Rey Juan Carlos (URJC), 28922 Alcorcón, Spain; 4 Department of Medical Gastroenterology, Mech-Sense, Aalborg University Hospital, 9000 Aalborg, Denmark

**Keywords:** single nucleotide polymorphism, inflammation, COVID-19, pain, post-COVID

## Abstract

Our aim was to assess the association between four inflammatory polymorphisms with the development of post-COVID pain and to associate these polymorphisms with the clinical pain phenotype in individuals who had been hospitalized by COVID-19. Three potential genotypes of IL-6 (rs1800796), IL-10 (rs1800896), TNF-α (rs1800629), and IFITM3 (rs12252) single nucleotide polymorphisms (SNPs) were obtained from no-stimulated saliva samples from 293 (49.5% female, mean age: 55.6 ± 12.9 years) previously hospitalized COVID-19 survivors by polymerase chain reactions. Pain phenotyping consisted of the evaluation of pain features, sensitization-associated symptoms, anxiety levels, depressive levels, sleep quality, catastrophizing, and kinesiophobia levels in patients with post-COVID pain. Analyses were conducted to associate clinical features with genotypes. One hundred and seventeen (39.9%) patients experienced post-COVID pain 17.8 ± 5.2 months after hospital discharge. No significant differences in the distribution of the genotype variants of any SNPs were identified between COVID-19 survivors with and without post-COVID pain (all, *p* > 0.47). Similarly, the clinical pain phenotype was not significantly different between patients with and without post-COVID pain since no differences in any variable were observed for any SNPs. In conclusion, four SNPs associated with inflammatory and immune responses did not appear to be associated with post-COVID pain in previously hospitalized COVID-19 survivors. Further, neither of the SNPs were involved in the phenotyping features of post-COVID pain.

## 1. Introduction

Clinical manifestations of the Severe Acute Respiratory Syndrome Coronavirus-2 (SARS-CoV-2), the virus causing the coronavirus disease 2019 (COVID-19), are heterogeneous and affect different systems [1]. In addition, current evidence is clear on the presence of symptoms after the acute phase of SARS-CoV-2 infection, a condition called long-COVID [2] or post-COVID-19 [3]. Long-COVID is a heterogeneous condition, and patients can experience a plethora of symptoms affecting multiple systems [4]. Among the heterogeneous symptoms reported by people with long-COVID, pain is an important and disabling symptom, showing a prevalence up to 25% during the first 6 months after the infection [5]. Different theories have been proposed for explaining the potential mechanisms associated with post-COVID pain. The most accepted hypothesis is that a prolonged pro-inflammatory response (i.e., cytokine storm) associated to SARS-CoV-2 acute infection provokes an exaggerated immune response leading to an hyperexcitability of the nervous system [6,7].

Several pro-inflammatory cytokines can be involved in the COVID-19 associated storm. Interleukin 6 (IL-6) is generated by different cells, e.g., fibroblasts and macrophages, as a response to tissue damage and disease [8]. In certain murine viral diseases, IL-6 has a protective role and contributes to the resolution of the acute infection, but, in others, including SARS-CoV-2, elevated concentrations of IL-6 are associated with pulmonary lesions [9], acute respiratory failure [10], and mortality [11]. In fact, overproduction of IL-6 is a hallmark of severe COVID-19 with a critical role in exacerbating the excessive inflammatory response of the host. Similarly, a dramatic increase in interleukin 10 (IL-10) is also seen in people with severe COVID-19 [12]. In fact, IL-10 concentrations strongly correlated with those of IL-6 and other inflammatory markers, e.g., C-reactive protein [13]. Han found that IL-6 and IL-10 levels could identify patients with higher risk of COVID-19 disease deterioration [14].

Tumor necrosis factor α (TNF-α) is another cytokine, secreted by macrophages and T-cells, that also plays a key role on inflammation, cell proliferation, differentiation, and apoptosis [15]. During acute SARS-CoV-2 infection, the TNF-α-derived inflammatory cascade also contributes to lung damage [13]. Finally, interferon induced transmembrane (IFITM) proteins are also important for antiviral protection in adaptive and innate immunity. Particularly, IFITM3 promotes mucosal immune longevity by increasing the concentration of CD8+T lymphocytes in the airways, a vital process in viral respiratory diseases [16,17]. In fact, IFITM3 has been associated with COVID-19 severity and a higher cytokine storm [18].

Different polymorphisms account for the variability of these cytokines’ expression in different infectious diseases. For example, IL-6 rs1800796 and IL-10 rs1800896 single nucleotide polymorphisms (SNPs) have been used to explain specific susceptibility to some respiratory viral infections. However, the role of IL-6 rs1800796 SNP in COVID-19 is controversial. Chen et al. identified that the variant allele C of this SNP, associated with lower IL-6 expression, showed a protective role against COVID-19 severity [19]; however, Falahi et al. did not observe such an association [20]. IL-10 SNPs have been also associated with severity and outcomes in patients with COVID-19. In fact, the variations in the prevalence of COVID-19 and its mortality among countries is explained by the IL-10 rs1800896 SNP [21]. Similarly, the A allele of the TNF-α rs1800629 SNP has been also associated with higher incidence of and more severe COVID-19 disease [22]. Nevertheless, a recent meta-analysis did not find an association between TNF-α rs1800629 and COVID-19 mortality [23]. Finally, previous research has shown that SNPs in the IFITM3 gene might reduce the antiviral activity of this protein, leading to an increase in infection sensitivity and disease severity. Moreover, a recent meta-analysis has confirmed that IFITM3 rs12252 SNP was associated with COVID-19 susceptibility [24].

Looking at the research, most studies investigating the role of these SNPs focused on the risk of being infected or the severity of the disease at the acute phase [19,20,21,22,23,24]. No study has previously investigated the role of cytokines’ polymorphisms on long-COVID symptoms. Considering the lack of evidence for the association between post-COVID pain and inflammatory/immune SNPs, it is now relevant to evaluate their associations to identify populations susceptible to experiencing long-term pain after SARS-CoV-2 infection and possibly for other similar potent viral infections. Therefore, our primary aim was to investigate the association between found SNPs, IL-6 (rs1800796), IL-10 (rs1800896), TNF-α (rs1800629), and IFITM3 (rs12252) and the presence of post-COVID pain in individuals previously hospitalized by COVID-19. Our secondary aim was to identify if any of the investigated SNPs were associated with the clinical phenotype in COVID-19 survivors specifically suffering from post-COVID pain.

## 2. Methods

### 2.1. Participants

This study included patients who were hospitalized due to COVID-19 at three urban hospitals in Madrid (Spain) during the first wave of the pandemic (March–May 2020). Participants had been diagnosed with an acute SARS-CoV-2 infection by real-time reverse transcription–polymerase chain reaction (RT-PCR) assay of a nasopharyngeal/oral swab sample and clinical and radiological findings at hospital admission. This study was approved by the Institutional Ethics Committees of all institutions/hospitals involved (HSO25112020; HUIL/092-20, HUFA 20/126, URJC0907202015920). All participants were informed of the study and all provided their written informed consent before collecting any data.

### 2.2. Defining Post-COVID Pain

Participants were scheduled for a face-to-face interview between 1 June and 31 November 2021 for collecting data. They were asked for the presence of pain symptoms that started at hospitalization or from days after hospitalization and persisted at the time of the assessment. We used the definition of musculoskeletal chronic pain proposed by the International Association for the Study of Pain (IASP) [25] for defining post-COVID pain. We focused on de novo pain symptoms starting after the acute infection and lasting for at least three consecutive months. Patients with history of pain symptoms before the infection or concomitant diagnosis of any pre-existing medical comorbidity better explaining pain symptoms were excluded.

### 2.3. DNA Collection and Genotyping

Unstimulated whole saliva samples were collected from each participant into collection tubes according to standardized procedures. Participants were asked not to eat, drink, or chew gum for 1 h before sample collection. Those who smoked were asked not to do so from 2 days before collection sampling. Immediately after the collection, the samples were centrifuged at 3000 rpm for 15 min to obtain the cell sediment and stored at −20 °C until the analysis.

The procedure of extraction was the same as a previous study [26]. Genomic DNA was extracted from 500 mL of saliva using a MagMAX™ DNA Multi-Sample Ultra 2.0 Kit (Thermo Fisher Scientific Inc, Hemel Hempstead, Hertfordshire, UK). We automatically extracted DNA using the King Fisher Flex purification robot (Thermo Fisher). The resulting DNA was assessed for purity and concentration using Quant-iT™ PicoGreen™ dsDNA reagent (Thermo Fisher). The resulting DNA was diluted to 5 ng/μL, using 1×Tris-EDTA (TE) buffer (Sigma-Aldrich, Dorset, UK). The qPCR reaction mixtures of 10 μL contained a total of 10 ng gDNA as a PCR template, 1× TaqMan Gene Expression PCR Master Mix, and 0.6× Genotyping TaqMan-probe assay.

For genotyping the single nucleotide polymorphisms with Real-Time PCR reaction (RT-PCR), TaqMan^®^ Predesigned SNP Genotyping Assays were used (Thermo Fisher Scientific Inc., Hemel Hempstead, Hertfordshire, UK). TaqMan^®^ SNP Genotyping Assays use TaqMan^®^ 5′-nuclease chemistry for amplifying and detecting specific polymorphisms in purified genomic DNA samples. Each assay allows the genotyping of individuals for a single nucleotide polymorphism (SNP). Real-time PCR plates were run in the Quantstudio 12 K Flex System (Thermo Fisher) of the Genomics Unit (Madrid Science Park Foundation, Spain) under standard running conditions (95 °C for 10 min and 40 2-step cycles consisting of 95 °C for 15 s and 60 °C for 1 min) and analyzed with the Genotyping App of Thermo Fisher Cloud. Identification of each of the three possible variants of each SNP was conducted by using specific fluorescent dyes.

Three possible genotypes (C/G, C/C, G/G) of the IL6 (rs1800796) polymorphism were derived from a C→G substitution, with the following sequence: ATGGCCAGGCAGTTCTACAACAGCC [C/G] CTCACAGGGGAGCCAGAACACAGA.

Three possible genotypes (C/C, T/C, T/T) of the IL10 (rs1800896) polymorphism were derived from a T→C substitution, with the following sequence: TCCTCTTACCTATCCCTACTTCCCC [T/C] TCCCAAAGAAGCCTTAGTAGTGTTG.

Three possible genotypes (A/A, A/G, G/G) of the TNF-α (rs1800629) polymorphism were derived from a A→G substitution, with the following sequence: GAGGCAATAGGTTTTGAGGGGCATG [A/G] GGACGGGGTTCAGCCTCCAGGGTCC.

Three possible genotypes (A/A, A/G, G/G) of the IFITM3 (rs12252) polymorphism were derived from a A→G substitution, with the following sequence: GCATCTCATAGTTGGGGGGCTGGCC [A/G] CTGTTGACAGGAGAGAAGAAGGTTT.

### 2.4. Post-COVID Pain Phenotyping

Individuals with post-COVID pain also fulfilled the following patient-reported outcome measures (PROMs) evaluating clinical, sensory-related, cognitive, and psychological variables.

Clinical variables: The intensity (numerical pain rating scale, NPRS, 0–10) and duration (years) of pain symptoms were self-reported.

Sensory-related variables: The Central Sensitization Inventory (CSI, 25-items, 5-point Likert scale, total score 0–100) evaluated the presence of sensitization-associated symptomatology [27], whereas the Self-Report Leeds Assessment of Neuropathic Symptoms (S-LANSS, 7-items, total score 0–24) identified the presence of neuropathic pain features [28].

Cognitive variables: Pain catastrophism was assessed with the Pain Catastrophizing Scale (PCS, 13-items, 4-point Likert scale, total score 0–52) [29], whereas kinesiophobia level was determined with the 11-item Tampa Scale Kinesiophobia Scale (TSK-11, 11-items, 4-point Likert scale, total score 11–44) [30].

Psychological variables: The Hospital Anxiety and Depression Scale (HADS-A, HADS-D, 7-items each, 3-point Likert scale, score 0–21 points) was used to evaluate anxiety/depressive levels [31], whereas the Pittsburgh Sleep Quality Index (PSQI, 19-items, total score 0–21 points) was used to assess the quality of sleep during the previous month [32].

### 2.5. Statistical Analysis

For the primary aim of the study, the Fisher exact test was used to detect differences in genotype frequencies of the SNPs between individuals with and without post-COVID pain. The chi-squared (χ2) test was used to assess deviations from the Hardy–Weinberg equilibrium. Differences in genotype frequencies in the total sample and between males and females were assessed with the Fisher exact test and chi-squared (χ2) tests.

For the secondary aim of the study, the Shapiro–Wilk test was used to assess the assumption of normality. Nonparametric tests were used if needed. Differences in sensory-related, cognitive, and psychological variables according to the genotypes of each SNP (IL6 rs1800796, IL-10 rs1800896, TNF -α rs1800629, IFITM3 rs12252) in those COVID-19 survivors with post-COVID pain symptoms were analyzed with a one-way analysis of variance (ANOVA). Post-hoc analyses were conducted with the Tukey test. All analyses were performed using SPSS Statistics, version 25.0. For all analyses, two-tailed tests were used and a *p*-value < 0.05 was considered significant.

## 3. Results

As previously described [26], 293 (49.5% female, age: 55.6 ± 12.9 years) previously hospitalized COVID-19 survivors from a sample of 350 invited to participate fulfilled all inclusion criteria. Fifty-seven (16%) were excluded: (1) refuse to participate (*n* = 30); (2) comorbid diagnosis of fibromyalgia (*n* = 20); (3) pain from neurological origin (*n* = 5); or (4) pregnancy (*n* = 2). At the time of study (mean 17.8, SD 5.2 months after hospital discharge), 117 (40%) patients fulfilled the criteria for musculoskeletal post-COVID pain.

Two saliva samples were compromised during genotyping analysis for the investigated SNPs and excluded from the analysis. Additionally, the genotype of some SNPs in the other two samples was indeterminate, leading to the following numbers on each SNP: IL6 rs1800796 (*n* = 291), IL-10 rs1800896 (*n* = 290), TNF-α rs1800629 (*n* = 290), and IFITM3 rs12252 (*n* = 290).

The genotype distributions did not deviate from that expected based on the Hardy–Weinberg equilibrium. No significant differences in the distribution of the genotypes of any SNP were found (IL6 rs1800796, *p* = 0.637; IL-10 rs1800896, *p* = 0.673; TNF-α rs1800629, *p* = 0.570; IFITM3 rs12252, *p* = 0.473) between COVID-19 survivors with and without post-COVID pain (Table 1). Similarly, no significant differences in the distribution of genotypes of any SNP (IL6 rs1800796, *p* = 0.967; IL-10 rs1800896, *p* = 0.519; TNF-α rs1800629, *p* = 0.350; IFITM3 rs12252, *p* = 0.417) between males and females were observed.

No significant differences in clinical, sensory-related, cognitive, and psychological/emotional variables according to the genotype for any SNP IL6 rs1800796 (Table 2), IL-10 rs1800896 (Table 3), TNF-α rs1800629 (Table 4), and IFITM3 rs12252 (Table 5) were identified based on the mixed-model ANOVA.

## 4. Discussion

This study did not find significant differences in the presence of IL-6 (rs1800796), IL-10 (rs1800896), TNF-α (rs1800629), and IFITM3 (rs12252) SNPs between COVID-19 survivors with and without post-COVID pain. Additionally, none of the analyzed SNPs were related to clinical phenotype of post-COVID pain.

Previous studies have looked thoroughly into the associations of SNPs and the predisposition to and severity of COVID-19 disease to identify individuals at risk [19,20,21,22,23,24]. To the best of our knowledge, this is the first study aiming to study the potential associations between SNPs related to SARS-CoV-2 related cytokine storm and post-COVID pain in individuals who had been previously hospitalized by COVID-19.

Potential underlying mechanisms proposed for explaining the development of post-COVID pain include systemic immune cell hyperactivation due to prolonged inflammation, viral toxicity, microvascular injury due to hypercoagulability, as well as psychological factors [33]. Several of the proinflammatory signaling molecules elevated in COVID-19 due to the cytokine storm impact the skeletal muscle nociception. Current evidence about differences in serological biomarkers of inflammation at the acute phase of the infection and the development of post-COVID pain is controversial. Two studies have found differences in creatine kinase or higher D-dimer levels at hospital admission between patients who developed post-COVID pain and those who did not [34,35]. On the contrary, another study identified that these differences in serological biomarker at hospital admission during the acute phase of SARS-CoV-2 infection were small and not relevant [36]. Similarly, a small proportion of COVID-19 survivors also have elevated D-dimer and C-reactive protein levels two months after hospitalization; however, no significant association with any post-COVID symptom was seen [37]. A pilot study including 15 patients showed a high prevalence of an antinuclear antibodies positive screen in COVID-19 patients exhibiting post-COVID joint pain [38]. These heterogeneous results could be related to a genetic influence, as differences in SNPs can regulate the levels of their associated biomarkers.

No previous study has investigated the role of selected inflammatory SNPs in the development of post-COVID pain. Current data about the role of IL6 rs1800796, IL-10 rs1800896, TNF-α rs1800629, and IFITM3 rs12252 SNPs are inconclusive. These SNPs have been associated with a higher risk of being infected and with a worse severity of COVID-19 disease in single studies; however, meta-analyses have reported conflicting results [19,20,21,22,23,24]. In the current study, any genotype associated with IL6 rs1800796, IL-10 rs1800896, TNF -α rs1800629, and IFITM3 rs12252 SNPs was significantly different between COVID-19 survivors with and without post-COVID pain, and, therefore, also with different pain phenotype features. This lack of difference does not exclude the role of the cytokine storm in long-COVID symptoms. These results suggest no influence of the genetic biomarkers investigated on the development of post-COVID pain, but we did not assess the presence of inflammation, per se, in our sample of long-COVID patients. It is possible that a prolonged disease duration, e.g., low levels of systemic inflammation, could be related to long-term post-COVID pain. In fact, it has been recently seen that prolonged systematic inflammation present after the acute phase of the infection is related to more post-COVID symptomatology and should be therapeutically targeted [39]. This assumption is based on a recent study showing that long-lasting systemic inflammation was present in people with more severe long COVID symptomatology one year after infection [40]. It is also possible that other, not investigated SNPs could be related to post-COVID pain rather than those investigated in the current study.

We should recognize some potential limitations of the study. First, our sample was only formed by individuals who had been previously hospitalized due to an acute SARS-CoV-2 infection; accordingly, the role of the investigated SNPs in non-hospitalized COVID-19 survivors is not currently known. Second, the sample size could be considered small, particularly for detecting sex differences. Population based cohort studies should be conducted to confirm or refute the results from this study. In fact, normal genetic variance in the general population can be seen in the literature. Third, we only analyzed for selected SNPs commonly shown to be associated with COVID-19 severity. It is possible that other biomarkers related to different aspects of the immune inflammatory regulation can reveal different results. Finally, collecting data was conducted in a period when some patients could have been vaccinated. We did not collect vaccination status. A recent systematic review concluded that the impact of vaccination in individuals with long-COVID symptoms is controversial, with some data showing changes in symptoms while others did not [41]. Accordingly, we believe that the impact of vaccines on SNPs would be minimal.

## 5. Conclusions

This study showed that four polymorphisms associated with inflammatory response (SNPs of IL-6 (rs1800796), IL-10 (rs1800896), TNF-α (rs1800629), and IFITM3 (rs12252)), were not associated with post-COVID pain in individuals previously hospitalized by COVID-19 at a follow-up period of 18 months. Additionally, the analyzed SNPs were not related to pain phenotype since no differences in clinical, sensory-related, cognitive and psychological variables were found. Larger population cohort studies investigating other specific polymorphisms and deeply analyzing sex differences could help to understand underlying pathophysiology and phenotyping of post-COVID pain.

## Figures and Tables

**Table 1:** Genotype distribution of the inflammatory polymorphisms in COVID-19 survivors with and without post-COVID pain.

	Post-COVID Pain	No Post-COVID	*p* Value
IL6 rs1800796 (*n* = 291)			
G/G (*n* = 226)	91 (77.7%)	135 (77.5%)	χ^2^ = 0.903; *p* = 0.637
C/G (*n* = 60)	23 (19.7%)	37 (21.3%)	
C/C (*n* = 5)	3 (2.6%)	2 (1.2%)	
IL-10 rs1800896 (*n* = 290)			
T/T (*n* = 112)	46 (39.3%)	66 (38.2%)	χ^2^ = 0.793; *p* = 0.673
T/C (*n* = 134)	51 (43.6%)	83 (47.9%)	
C/C (*n* = 44)	20 (17.1%)	24 (13.9%)	
TNF-α rs1800629 (*n* = 290)			
G/G (*n* = 229)	95 (81.2%)	134 (77.5%)	χ^2^ = 1.125; *p* = 0.570
A/G (*n* = 55)	19 (16.2%)	36 (20.8%)	
A/A (*n* = 6)	3 (2.6%)	3 (1.7%)	
IFITM3 rs12252 (*n* = 290)			
A/A (*n* = 243)	95 (81.2%)	148 (85.5%)	χ^2^ = 1.499; *p* = 0.473
A/G (*n* = 44)	20 (17.1%)	24 (13.9%)	
G/G (*n* = 3)	2 (1.7%)	1 (0.6%)	

**Table 2:** Differences in pain phenotyping in COVID-19 survivors with post-COVID pain depending on the Interleukin-6 (IL-6) polymorphism (rs1800796).

	G/G (*n* = 91)	C/G (*n* = 23)	C/C (*n* = 3)
Demographic Features			
Age (years)	55.3 ± 12.5	55.6 ± 11.9	55.0 ± 7.8
Height (m)	1.63 ± 0.2	1.65 ± 0.1	1.63 ± 0.1
Weight (kg)	81.4 ± 17.6	79.3 ± 20.3	80.7 ± 7.5
Clinical Features			
Pain intensity (NPRS, 0–10)	5.7 ± 1.6	5.4 ± 2.0	5.0 ± 1.8
Post-COVID Symptoms (months)	17.9 ± 4.83	17.6 ± 5.9	16.3 ± 6.1
Sensory-Related Variables			
CSI (0–100)	35.0 ± 18.7	34.5 ± 15.7	40.2 ± 16.4
S-LANSS (0–24)	7.2 ± 6.7	6.3 ± 5.9	8.3 ± 2.5
Cognitive Variables			
PCS (0–52)	8.6 ± 7.8	7.0 ± 6.7	8.3 ± 3.4
TSK-11 (0–44)	22.7 ± 8.5	23.8 ± 9.8	26.0 ± 7.5
Psychological Variables			
HADS-A (0–21)	4.7 ± 4.1	4.8 ± 4.9	4.3 ± 6.0
HADS-D (0–21)	3.7 ± 4.2	4.8 ± 5.0	4.4 ± 3.0
PSQI (0–21)	7.6 ± 3.9	6.7 ± 3.2	7.0 ± 7.1

NPRS: Numerical Pain Rate Scale; CSI: Central Sensitization Inventory; S-LANSS: self-reported version of the Leeds Assessment of Neuropathic Symptoms and Signs; PCS: Pain Catastrophizing Scale; TSK-11: 11-items Tampa Scale for Kinesiophobia; HADS: Hospital Anxiety and Depression Scale (A: Anxiety; D: Depression PSQI: Pittsburgh Sleeping Quality Index).

**Table 3:** Differences in pain phenotyping in COVID-19 survivors with post-COVID pain depending on the Interleukin-10 (IL-6) polymorphism (rs1800896).

	T/T (*n* = 46)	T/C (*n* = 51)	C/C (*n* = 20)
Demographic Features			
Age (years)	53.9 ± 12.2	57.1 ± 12.6	52.7 ± 11.8
Height (m)	1.65 ± 0.1	1.64 ± 0.07	1.69 ± 0.1
Weight (kg)	80.7 ± 16.9	79.1 ± 11.0	82.9 ± 15.7
Clinical Features			
Pain intensity (NPRS, 0–10)	5.3 ± 1.4	5.9 ± 1.9	5.5 ± 1.6
Post-COVID Symptoms (months)	18.5 ± 4.7	16.8 ± 5.9	18.1 ± 3.8
Sensory-Related Variables			
CSI (0–100)	38.7 ± 18.6	34.6 ± 18.4	37.8 ± 17.3
S-LANSS (0–24)	7.0 ± 6.5	7.8 ± 6.2	7.9 ± 4.3
Cognitive Variables			
PCS (0–52)	9.9 ± 9.5	8.8 ± 8.0	8.7 ± 6.5
TSK-11 (0–44)	25.1 ± 9.5	23.5 ± 8.1	23.4 ± 6.8
Psychological Variables			
HADS-A (0–21)	5.1 ± 4.4	4.6 ± 4.1	4.7 ± 4.7
HADS-D (0–21)	4.1 ± 4.3	4.1 ± 4.4	4.3 ± 4.1
PSQI (0–21)	7.5 ± 3.6	7.6 ± 3.9	7.3 ± 4.5

NPRS: Numerical Pain Rate Scale; CSI: Central Sensitization Inventory; S-LANSS: self-reported version of the Leeds Assessment of Neuropathic Symptoms and Signs; PCS: Pain Catastrophizing Scale; TSK-11: 11-items Tampa Scale for Kinesiophobia; HADS: Hospital Anxiety and Depression Scale (A: Anxiety; D: Depression PSQI: Pittsburgh Sleeping Quality Index).

**Table 4:** Differences in pain phenotyping in COVID-19 survivors with post-COVID pain depending on the Tumor Necrosis Factor α (TNF-α) polymorphism (rs1800629).

	G/G (*n* = 95)	A/G (*n* = 19)	A/A (*n* = 3)
Demographic Features			
Age (years)	55.1 ± 12.1	54.7 ± 14.7	56.0 ± 4.5
Height (m)	1.63 ± 0.1	1.64 ± 0.1	1.68 ± 0.15
Weight (kg)	80.0 ± 16.2	81.0 ± 13.9	89.3 ± 15.5
Clinical Features			
Pain intensity (NPRS, 0–10)	5.7 ± 1.8	5.4 ± 1.1	5.0 ± 0.9
Post-COVID Symptoms (months)	17.4 ± 5.2	18.8 ± 4.9	19.0 ± 1.7
Sensory-Related Variables			
CSI (0–100)	34.6 ± 19.0	39.9 ± 14.3	40.3. ± 14.9
S-LANSS (0–24)	7.7 ± 8.7	8.0 ± 5.8	7.6 ± 6.4
Cognitive Variables			
PSC (0–52)	8.4 ± 10.2	7.9 ± 6.0	8.7 ± 6.5
TSK-11 (0–44)	23.3 ± 8.9	22.1 ± 7.8	25.3 ± 10.0
Psychological Variables			
HADS-A (0–21)	4.6 ± 4.4	4.1 ± 4.0	3.8 ± 2.1
HADS-D (0–21)	4.1 ± 4.5	4.0 ± 3.6	3.7 ± 1.2
PSQI (0–21)	7.5 ± 3.9	8.0 ± 3.5	7.0 ± 3.6

NPRS: Numerical Pain Rate Scale; CSI: Central Sensitization Inventory; S-LANSS: self-reported version of the Leeds Assessment of Neuropathic Symptoms and Signs; PCS: Pain Catastrophizing Scale; TSK-11: 11-items Tampa Scale for Kinesiophobia; HADS: Hospital Anxiety and Depression Scale (A: Anxiety; D: Depression PSQI: Pittsburgh Sleeping Quality Index).

**Table 5:** Differences in pain phenotyping in COVID-19 survivors with post-COVID pain depending on the Interferon Induced Transmembrane 3 (IFITM3) polymorphism (rs12252).

	A/A (*n* = 95)	A/G (*n* = 20)	G/G (*n* = 2)
Demographic Features			
Age (years)	55.9 ± 11.9	53.4 ± 14.2	54.5 ± 10.4
Height (m)	1.66 ± 0.07	1.63 ± 0.07	1.60 ± 0.06
Weight (kg)	80.1 ± 18.4	83.7 ± 17.0	78.0 ± 10.1
Clinical Features			
Pain intensity (NPRS, 0–10)	5.7 ± 1.6	5.4 ± 1.8	5.3 ± 0.5
Post-COVID Symptoms (months)	17.9 ± 5.3	17.0 ± 4.9	17.5 ± 3.5
Sensory-Related Variables			
CSI (0–100)	34.9 ± 18.4	37.2 ± 17.9	39.5 ± 11.9
S-LANSS (0–24)	7.0 ± 9.4	7.9 ± 7.5	7.0 ± 1.4
Cognitive Variables			
PCS (0–52)	7.7 ± 8.1	9.1 ± 11.1	12.5 ± 11.8
TSK-11 (0–44)	22.6 ± 8.2	24.6 ± 10.4	25.5 ± 4.9
Psychological Variables			
HADS-A (0–21)	5.0 ± 4.5	3.65 ± 3.3	5.5 ± 0.7
HADS-D (0–21)	4.1 ± 3.5	4.3 ± 3.7	4.5 ± 3.5
PSQI (0–21)	7.8 ± 3.9	7.7 ± 2.9	9.5 ± 2.1

NPRS: Numerical Pain Rate Scale; CSI: Central Sensitization Inventory; S-LANSS: self-reported version of the Leeds Assessment of Neuropathic Symptoms and Signs; PCS: Pain Catastrophizing Scale; TSK-11: 11-items Tampa Scale for Kinesiophobia; HADS: Hospital Anxiety and Depression Scale (A: Anxiety; D: Depression PSQI: Pittsburgh Sleeping Quality Index).

## Data Availability

All data derived from this study are presented in the text.
